# A Case Report of IgA Nephropathy Coexisting with Focal Segmental Glomerulosclerosis

**DOI:** 10.7759/cureus.5040

**Published:** 2019-06-29

**Authors:** Hassaan Iftikhar, Maryam Saleem, Anand Kaji

**Affiliations:** 1 Internal Medicine, St. Francis Medical Center, Seton Hall University, Trenton, USA; 2 Internal Medicine, Waterbury Hospital, Waterbury, USA

**Keywords:** fsgs, iga, nephropathy, glomerulonephritis

## Abstract

IgA nephropathy is a condition characterized by deposition of IgA immunoglobulin in the glomerulus. This condition is fairly common in Western countries. The disease spectrum is broad and it varies case to case. The cases of IgA nephropathy associated with focal segmental glomerulosclerosis are rare. Our case report is about a young male who developed rapid onset IgA nephropathy leading to end stage renal disease (ESRD).

## Introduction

IgA nephropathy is the most common cause of glomerulonephritis in the developed world [1]. However, cases of IgA nephropathy presenting with focal segmental glomerulosclerosis (FSGS) leading to end stage renal disease (ESRD) are exceedingly rare. In this case report, we describe a young male who presented with microscopic hematuria, severe proteinuria, and ended up on hemodialysis due to burnt out IgA nephropathy associated with FSGS.

## Case presentation

This is a 26-year-old male with no past medical history who presented with bilateral limb swelling and scrotal swelling for last two weeks. He was feeling more fatigued than usual and had experienced few episodes of vomiting over the same time course. He denied any fever, chills, recent sore throat, skin rash, changes in urine character, or recent travel history. He was not on any home medications. He was a lifetime non-smoker and had no history of polysubstance abuse. The family history was significant for hypertension in mother.

His physical exam on presentation was significant for blood pressure of 210/100 mmHg and other vital signs were within normal limits. He had swollen scrotum with no tenderness to palpation, and 3+ bilateral lower extremity edema extending up to the thighs. There was no pharyngeal exudate, renal bruit, or abnormal skin rash.

His major laboratory findings are described in Tables 1-3 below.

**Table 1:** Serum chemistry

Test	Result	Normal value
Serum Chemistry		
Sodium	139 mmol/L	136-145 mmol/L
Potassium	4.2 mmol/L	3.4-4.7 mmol/L
Chloride	106 mmol/L	98-107 mmol/L
Bicarbonate	25 mmol/L	21-31 mmol/L
Blood urea nitrogen (BUN)	60 mg/dl	7-25 mg/dl
Creatinine (Cr)	9.85 mg/dl	0.7-1.3 mg/dl
Albumin	2.6 g/dl	3.5-5.0 g/dl
Total protein	4.9 g/dl	6.4-8.3 g/dl

**Table 2:** Hematology

Hematology	Result	Normal value
Hemoglobin (Hb)	8.2 g/dl	12-16 g/dl
Platelets	71,000 thou/L	130-400 thou/L
White blood cells	8600 cells/uL	4.8-10.8 cells/uL
Mean corpuscular volume	97.5 fL	78-102 fL

**Table 3:** Lipid panel

Lipid panel	Result	Normal value
Cholesterol	209 mg/dl	<200 mg/dl
Low density lipid (LDL)	111 mg/dl	<129 mg/dl
Triglycerides (TG)	113 mg/dl	<150 mg/dl
High density lipid (HDL)	58 mg/dl	>40 mg/dl

His dipstick urinalysis was significant for large blood, >1000 mg/dl protein, and 10-20 red blood cells (RBCs) on microscopy. His urine studies revealed protein-to-creatinine ratio of 4.26 g/dl. Given his nephrotic range proteinuria, extensive workup was performed to uncover the etiology for nephrotic syndrome. His coagulation profile was normal, but his cholesterol was elevated (Table 3) and he had hypoalbuminemia (Table 1). His C3 was low at 72 mg/dl (normal range 98-140 mg/dl) with normal C4. Serum and urine protein electrophoresis were performed, which indicated hypogammaglobulinemia but no abnormal protein spike. Anti-nuclear antibody and anti-glomerular basement membrane antibody (anti-GBM) were negative and antistreptolysin O (ASO) titers were within normal limits. HIV, rapid plasma reagin, and hepatitis panel were negative. Cytoplasmic-antineutrophil cytoplasmic antibody (C-ANCA) and P-ANCA antibodies were negative as well. Glycosylated hemoglobin (HbA1C) was 4.2%. The computerized tomography scan (CT) of abdomen/pelvis and ultrasound of kidneys both demonstrated increased renal echogenicity. Serum iron panel revealed picture of anemia of chronic disease with ferritin of 294 mg/dl and serum iron saturation of <10%.

**Figure 1:**
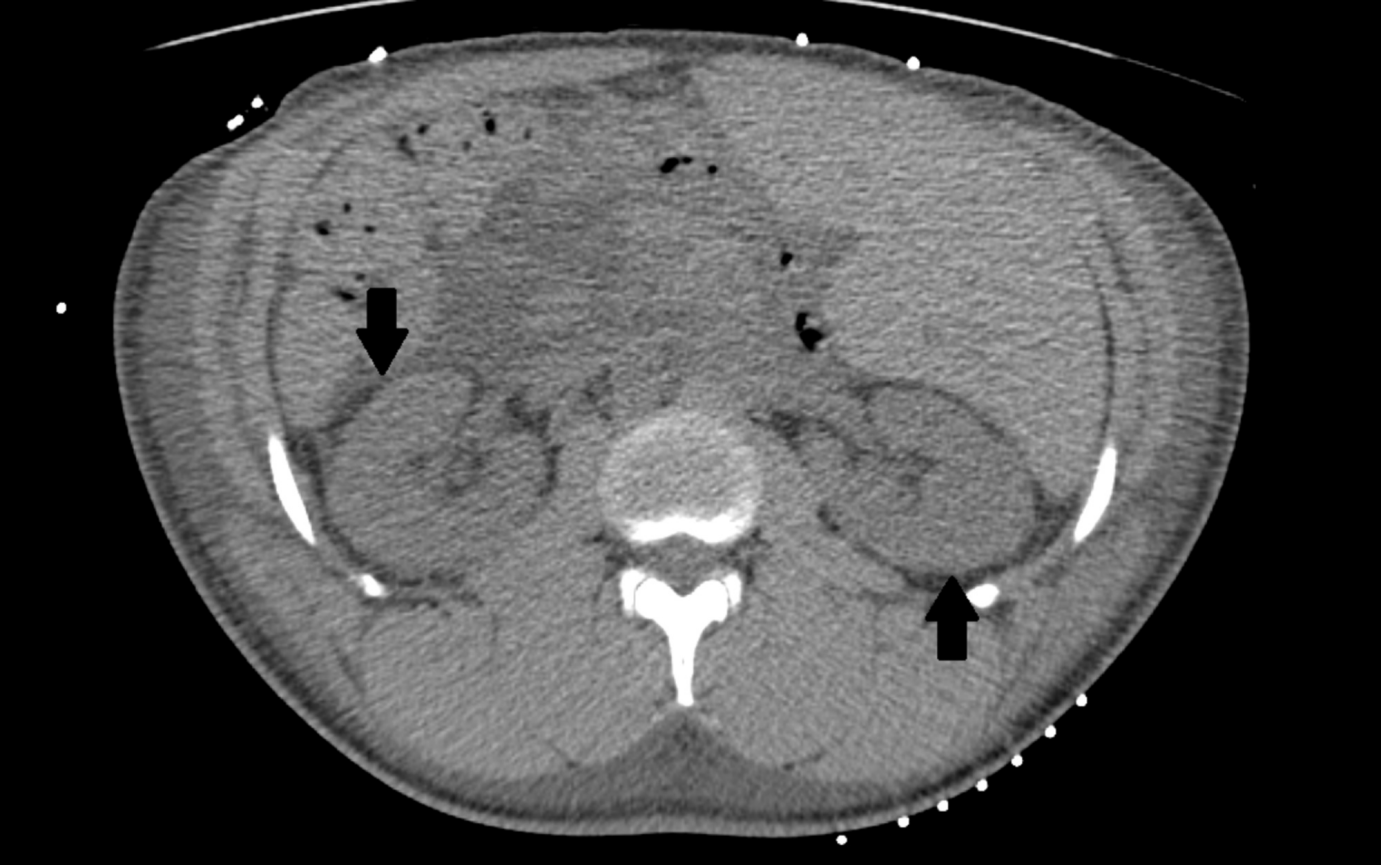
CT scan abdomen and pelvis Black arrows point towards increased echogenicity in bilateral renal cortex.

The CT scan of kidneys is shown in Figure 1 below.

**Figure 2:**
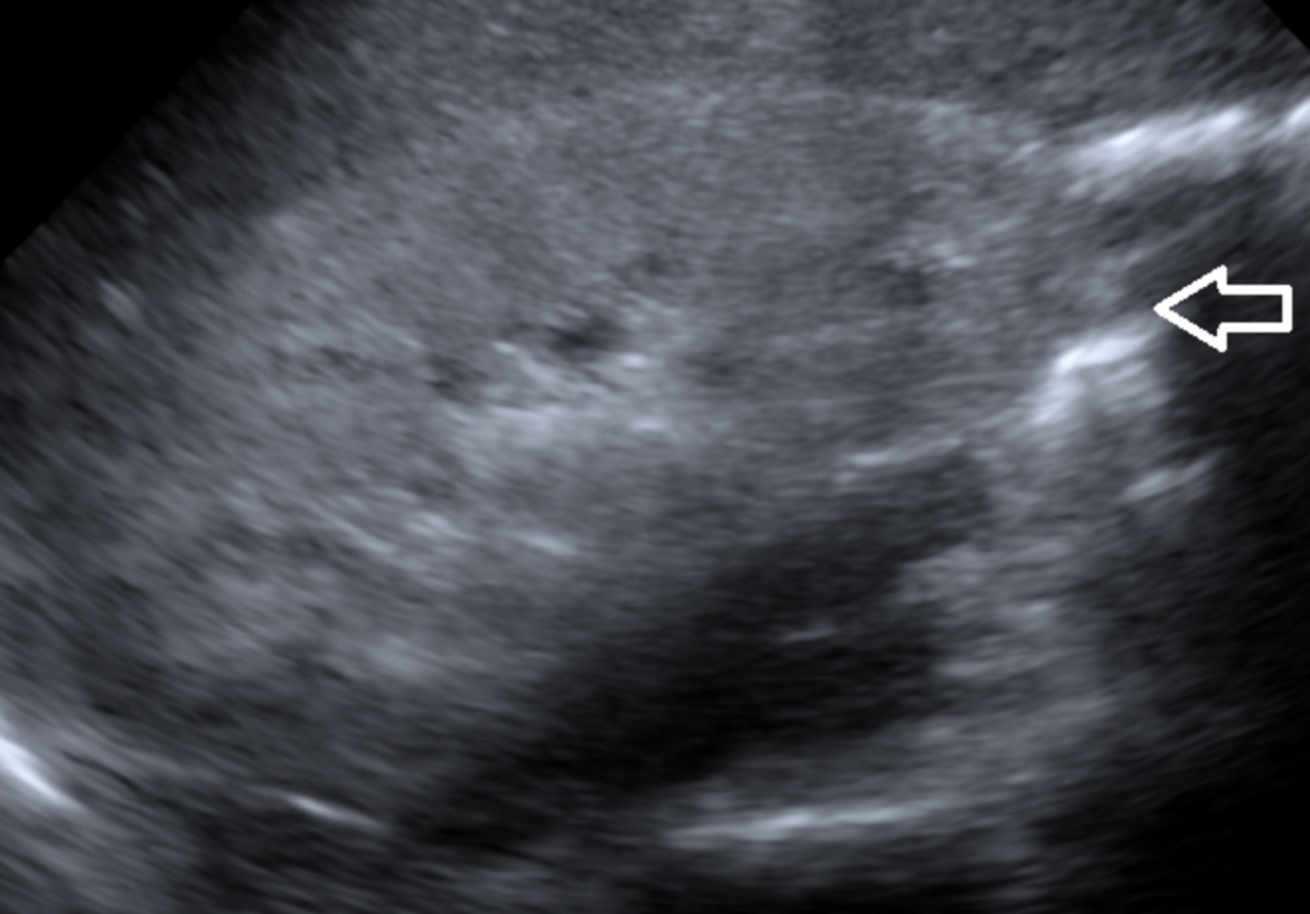
Ultrasound right kidney White arrow points towards increased echogenicity in renal cortex.

The ultrasound image of right kidney is shown in Figure 2 below.

Given his presentation with hypertensive urgency, causes for secondary hypertension were explored as well. The patient was found to have normal plasma metanephrine and normetanephrine levels, and plasma renin to aldosterone ratio and plasma aldosterone levels were also unremarkable.

During the course of the hospitalization, he was started on nicardipine drip for aggressive blood pressure control and later switched to oral anti-hypertensive medications, including carvedilol, hydralazine, isosorbide dinitrate, and diltiazem. His urine output throughout the hospital course stayed less than 500 ml/day, i.e., he was oliguric. He was also started on furosemide, which did not improve his urine output. His lower extremity and scrotal edema only improved slightly. He was scheduled for renal biopsy to discover the accurate etiology for the nephrotic syndrome. He was also started on intermittent hemodialysis (HD) as his creatinine increased to 12.45 mg/dl with blood urea nitrogen (BUN) to 88 mg/dl. His edema improved significantly with HD and the uremia resolved. His anemia was treated with intravenous iron and erythropoietin-stimulating agents. His platelet counts also improved significantly once his blood pressure was better controlled and it was postulated to be decreased because of thrombotic microangiopathy in setting of malignant hypertension.

**Figure 3:**
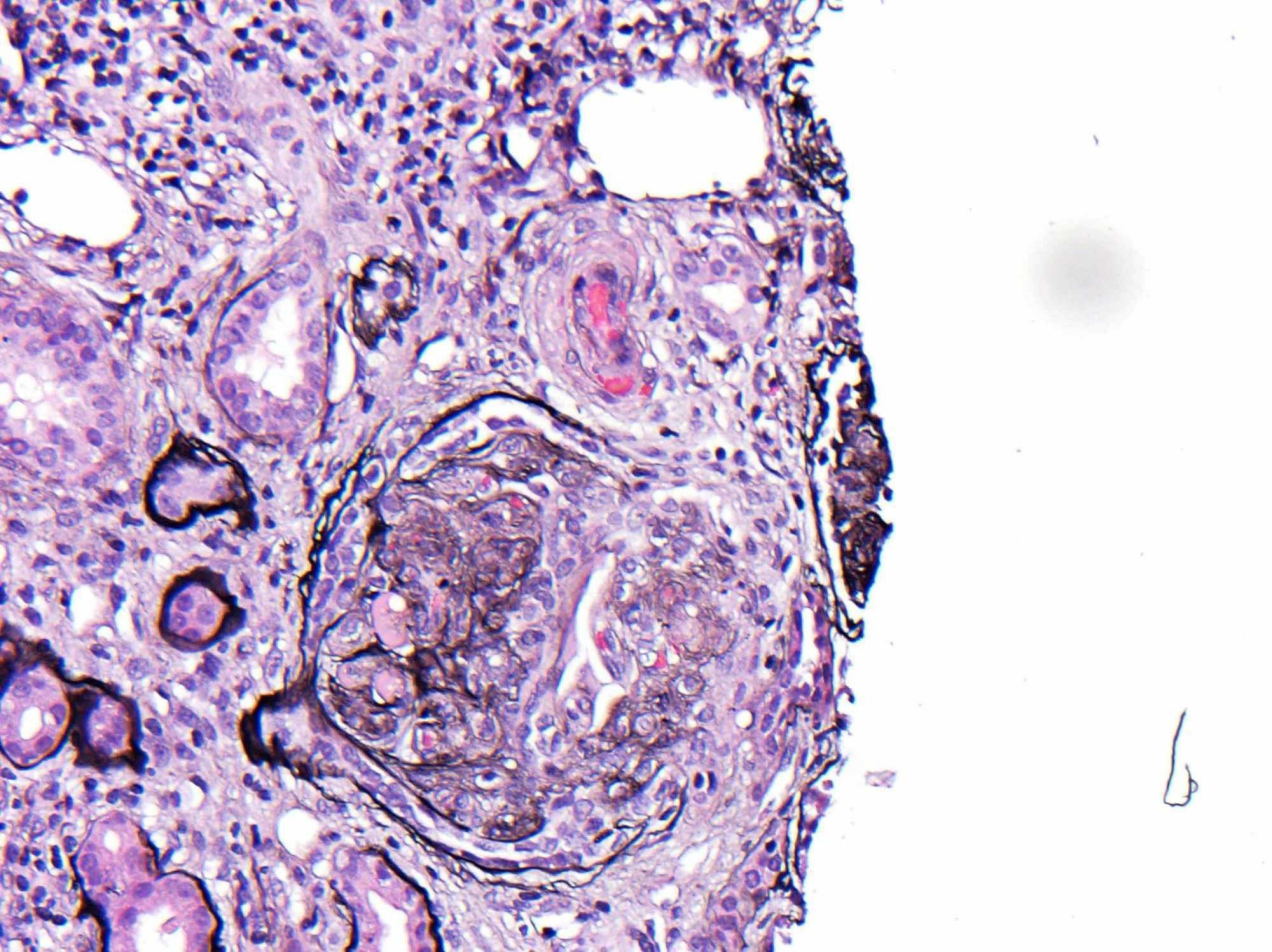
Light microscopy shows glomerular sclerosis

On biopsy, light microscopy (LM) showed that specimen had 29 globally sclerotic glomeruli and up to three additional glomeruli per level of section had segmental sclerosis with hyalinosis and two partial cellular crescents as shown in Figure 3.

**Figure 4:**
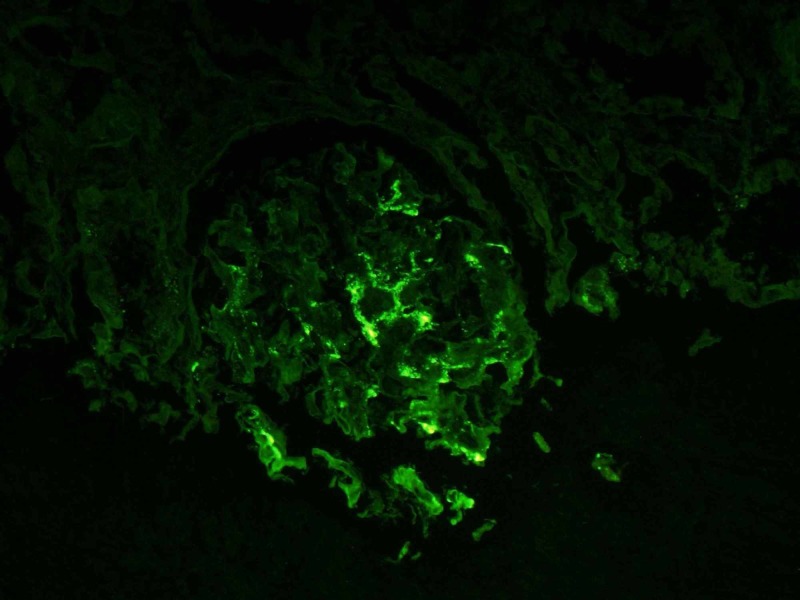
Positive immunofluorescence for lambda chains

**Figure 5:**
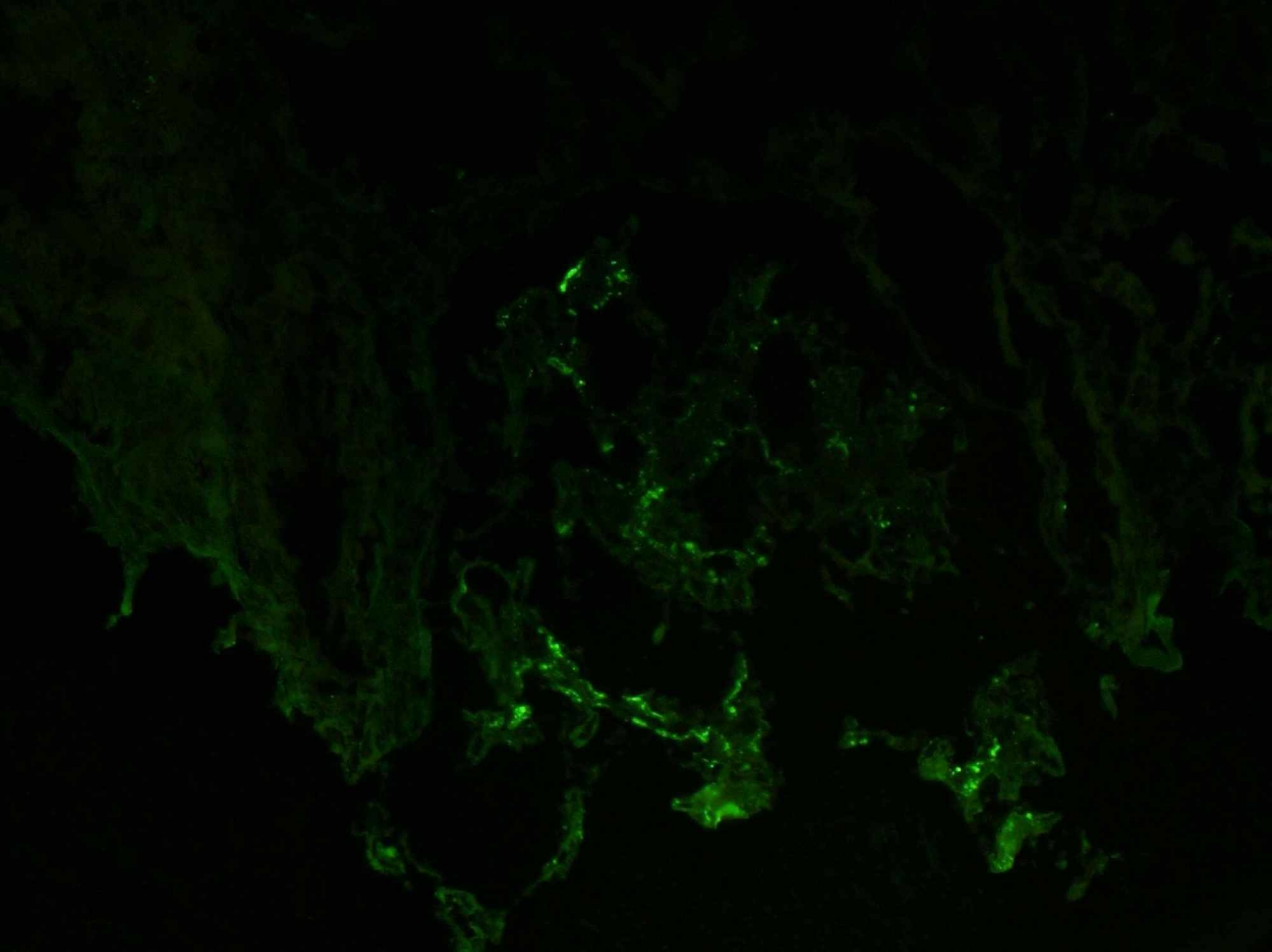
Positive immunofluorescence for IgA

For immunofluorescence (IF), hematoxylin and eosin stains were used which revealed that five globally sclerotic glomeruli had granular segmental mesangial staining with antisera specific for IgA, C3, and Kappa and Lambda light chains but negative for C1q, IgM, and IgG antibodies as shown in Figures 4, 5.

**Figure 6:**
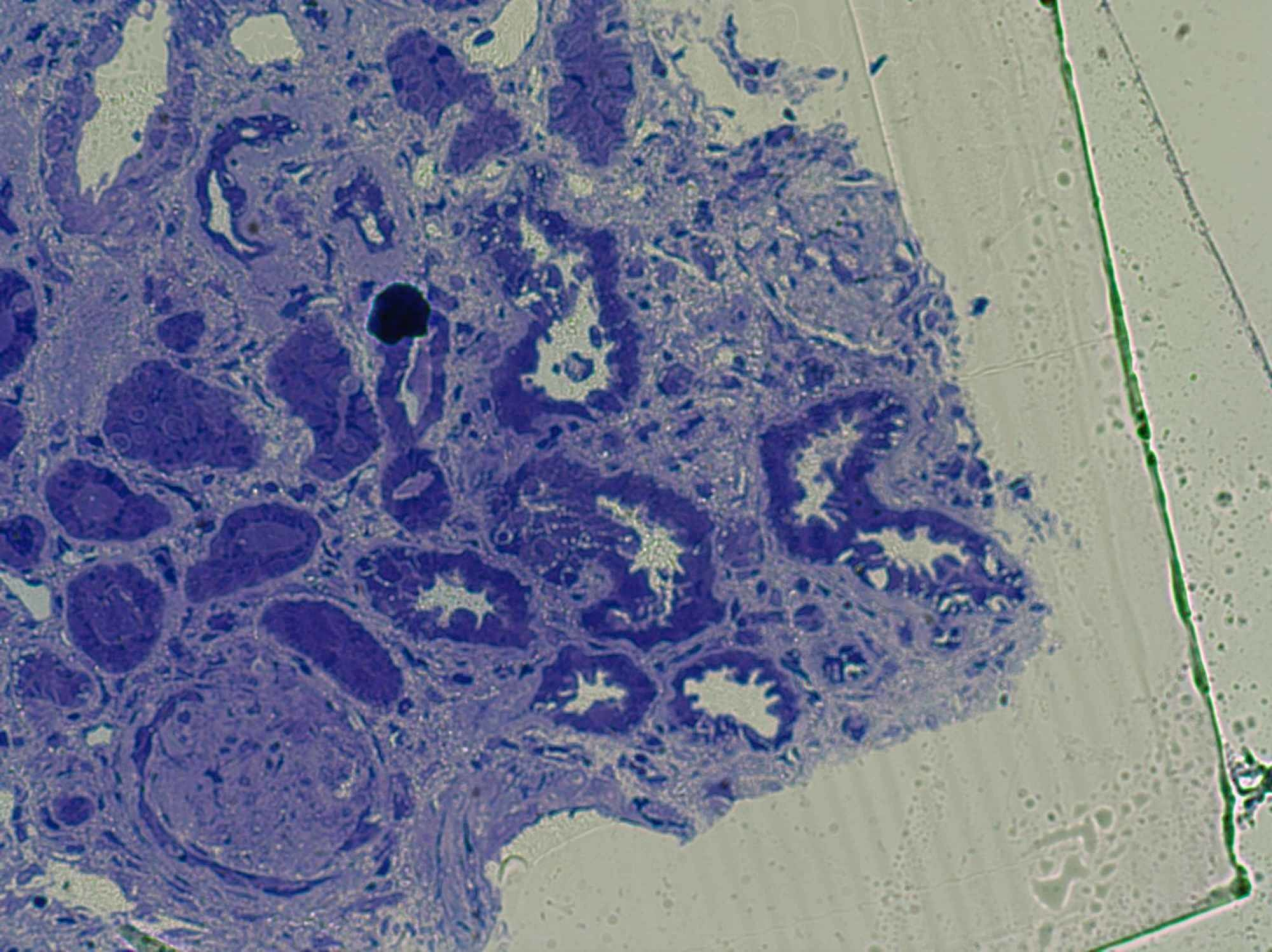
Electron microscopy shows global glomerular sclerosis

Electron microscopy in Figure 6 shows one globally sclerotic glomerulus.

Based on the biopsy results, he was diagnosed to have advanced diffuse sclerosing and proliferative IgA nephropathy with two partial cellular crescents and FSGS (2009 Oxford classification M1,E1,S1,T2) [2].

He was discharged on a beta-blocker, nondihydropyridine calcium-channel blocker, angiotensin-receptor-blocker, and hydralazine for aggressive blood pressure control and high-intensity statin therapy for hyperlipidemia. He was set up for hemodialysis three days a week with placement of arteriovenous fistula in the near future. He was also arranged to meet with transplant nephrologist for evaluation of renal transplant in the future.

## Discussion

IgA nephropathy has been described in the literature as being the top cause of glomerulonephritis, which is especially true for developed countries with the only exception being in Sub-Saharan Africa where it is found in only 1% of black patients with primary glomerular disease [1,3-5]. The incidence increases significantly around the second and third decades of life [6]. The cases of FSGS associated with IgA nephropathy have not been commonly reported in the literature and <10% of these patients have nephrotic syndrome progressing to hemodialysis [1,7]. The pathophysiology is not fully understood but it is known that deposition of IgA with a predominance of Lambda light chains occurs in the glomerular mesangium [5,8]. Glomerular deposition of C3 is also common but C1q is usually absent. Compared to healthy subjects, diseased patients have aberrational glycosylated IgA1 molecules in serum [5,8-10].

Patients usually present either as having gross hematuria after upper respiratory tract infection or may present as microscopic hematuria with varying degree of proteinuria [7]. Nephrotic-range proteinuria is seen in usually more severe cases. Thrombotic microangiopathy can also be found in cases of IgA nephropathy due to malignant hypertension and indicates a poor renal outcome as seen in our case [11].

For diagnostic purposes, renal biopsy is usually performed to stage the disease according to Oxford Classification, which also helps in determining the disease prognosis at the time of biopsy [2,12]. Clinical and laboratory findings at the time of diagnosis can also help stratify the severity of disease and these markers include increased serum creatinine (Cr), reduced glomerular filtration rate (GFR), hypertension (blood pressure > 140/90 mmHg) and persistent proteinuria [13,14]. The presence of the aforementioned features indicates a worse prognosis. On biopsy, if pathological findings of crescents, glomerular and/or segmental sclerosis, tubular atrophy, interstitial fibrosis, and interstitial cellular infiltrates are discovered, there is a worsening of disease state and elevated risk of developing ESRD [15,16]. For cases of IgA nephropathy with FSGS, there has been reported faster decline in GFR compared to IgA nephropathy alone [17].

There have only been observational studies for the treatment of IgA nephropathy with crescentic glomerulonephritis. It has not been evaluated in randomized controlled trials. The observational studies suggest the utilization of pulse intravenous (IV) methylprednisolone followed by oral prednisone, IV cyclophosphamide or plasmapheresis [18]. However, our patient was not given a trial of these therapies as he already had developed ESRD by the time of diagnosis. Non-immunosuppressive treatment options include utilization of angiotensin-converting enzyme inhibitors (ACE-I) or angiotensin receptor blockers (ARB) for better blood pressure control and proteinuria. A clinical trial suggested the use of ACE-I/ARB is superior to any other anti-hypertensive agent for such patients [19]. The monitoring of disease activity can be performed by regular assessment of GFR, serum Cr, proteinuria, and urinary sediment.

## Conclusions

Our case highlights the concurrent existence of IgA nephropathy with FSGS and indicates that co-existence leads to poor prognosis. There is a strong possibility of patients having a progressive course leading to ESRD and early diagnosis and treatment are of key importance.

## References

[1] Jennette JC (2006). Immunoglobulin A nephropathy and Henoch-Schönlein purpura. Fundamentals of Renal Pathology.

[2] Cattran DC, Coppo R, Cook HT (2009). The Oxford classification of IgA nephropathy: rationale, clinicopathological correlations, and classification. Kidney Int.

[3] Akkad I, Ortiz A, Hecht M (2016). Rapidly progressive IgA nephropathy: a case report with review of clinical presentation, prognostic factors and therapeutic modalities. J Med Cases.

[4] Seedat YK, Nathoo BC, Parag KB, Naiker IP, Ramsaroop R (1988). IgA nephropathy in Blacks and Indians of Natal. Nephron.

[5] Roberts IS (2014). Pathology of IgA nephropathy. Nat Rev Nephrol.

[6] Simon P, Ramee MP, Boulahrouz R (2004). Epidemiologic data of primary glomerular diseases in western France. Kidney Int.

[7] Donadio JV, Grande JP (2002). IgA nephropathy. N Engl J Med.

[8] Mestecky J, Tomana M, Moldoveanu Z (2008). Role of aberrant glycosylation of IgA1 molecules in the pathogenesis of IgA nephropathy. Kidney Blood Press Res.

[9] Lai KN (2012). Pathogenesis of IgA nephropathy. Nat Rev Nephrol.

[10] Suzuki H, Fan R, Zhang Z (2009). Aberrantly glycosylated IgA1 in IgA nephropathy patients is recognized by IgG antibodies with restricted heterogeneity. J Clin Invest.

[11] Chang A, Kowalewska J, Smith KD, Nicosia RF, Alpers CE (2006). A clinicopathologic study of thrombotic microangiopathy in the setting of IgA nephropathy. Clin Nephrol.

[12] Neelakantappa K, Gallo GR, Baldwin DS (1988). Proteinuria in IgA nephropathy. Kidney Int.

[13] Wakai K, Kawamura T, Endoh M (2006). A scoring system to predict renal outcome in IgA nephropathy: from a nationwide prospective study. Nephrol Dial Transplant.

[14] Lv J, Yang Y, Zhang H (2013). Prediction of outcomes in crescentic IgA nephropathy in a multicenter cohort study. J Am Soc Nephrol.

[15] Coppo R, Troyanov S, Camilla R (2010). The Oxford IgA nephropathy clinicopathological classification is valid for children as well as adults. Kidney Int.

[16] Sengul E, Eyileten T, Ozcan A, Yilmaz MI, Yenicesu M (2009). A case of crescentic IgA nephropathy treated with prednisolone and cyclophosphamide. Hippokratia.

[17] Weber CL, Rose CL, Magil AB (2009). Focal segmental glomerulosclerosis in mild IgA nephropathy: a clinical-pathologic study. Nephrol Dial Transplant.

[18] Tumlin JA, Lohavichan V, Hennigar R (2003). Crescentic, proliferative IgA nephropathy: clinical and histological response to methylprednisolone and intravenous cyclophosphamide. Nephrol Dial Transplant.

[19] Praga M, Gutiérrez E, González E, Morales E, Hernández E (2003). Treatment of IgA nephropathy with ACE inhibitors: a randomized and controlled trial. J Am Soc Nephrol.

